# Effects of oxygen on the antigenic landscape of prostate cancer cells

**DOI:** 10.1186/s13104-015-1633-7

**Published:** 2015-11-18

**Authors:** Tangeng Ma, Claire A. Schreiber, Gaylord J. Knutson, Abdelouahid El Khattouti, Marcelo J. Sakiyama, Mohamed Hassan, Mary Christine Charlesworth, Benjamin J. Madden, Xinchun Zhou, Stanimir Vuk-Pavlović, Christian R. Gomez

**Affiliations:** Cancer Institute, University of Mississippi Medical Center, 2500 North State Street, Jackson, MS 39216 USA; Department of Pathology, University of Mississippi Medical Center, 2500 North State Street, Jackson, MS 39216 USA; Department of Radiation Oncology, University of Mississippi Medical Center, 2500 North State Street, Jackson, MS 39216 USA; Stem Cell Laboratory, Mayo Clinic, 200 First St. SW, Rochester, MN 55905 USA; Proteomics Research Center, Mayo Clinic, 200 First St. SW, Rochester, MN 55905 USA; Division of Hematology, Department of Internal Medicine, Mayo Clinic, 200 First St. SW, Rochester, MN 55905 USA; Division of Preventive and Occupational Medicine, Department of Internal Medicine, Mayo Clinic, 200 First St. SW, Rochester, MN 55905 USA

**Keywords:** Tumor-associated antigens, Prostate cancer, Hypoxia, HSP60, HSP70, hnRNP L

## Abstract

**Background:**

Use of allogeneic cancer cells-based immunotherapy for treatment of established prostate cancer (PCa) has only been marginally effective. One reason for failure could stem from the mismatch of antigenic signatures of vaccine cells and cancer in situ. Hence, it is possible that vaccine cells expressed antigens differently than tumor cells in situ. We hypothesized that cells grown in vitro at low oxygen tension (*p*O_2_) provide a better antigen match to tumors in situ and could reveal a more relevant antigenic landscape than cells grown in atmospheric *p*O_2_.

**Methods:**

We tested this hypothesis by comparing PCa cells propagated at *p*O_2_ = 2 kPa and 20 kPa. To identify potential tumor-associated antigens (TAAs), we prepared PCa cell lysates, resolved them by two-dimensional electrophoresis and immunoblotting using spontaneous antibodies from plasma derived from PCa patients and control subjects. Antibody-labeled spots were analyzed by MALDI-TOF mass spectrometry and validated by ELISA. We selected hypoxia-regulated HSP70 and hnRNP L and hypoxia-independent HSP60 and determined the frequency of plasma samples reacting with these molecules.

**Results:**

Frequency of HSP60-reactive plasma was low in healthy controls [1.3 % (1/76)], while it was elevated in PCa patients [13.0 % (7/54); p < 0.05]. These data suggest a humoral immune response to HSP60 in PCa. Levels of autoantibodies to HSP70 did not differ from healthy controls [3.7 % (2/54)] in PCa patients [5.3 % (2/38)]. Similarly, hnRNP L autoantibodies did no differ between healthy controls [6.1 % (3/49)] and PCa patients [5.3 % (2/38)].

**Conclusions:**

Overall our results suggest the value of hypoxia as a modifier of the cellular and antigenic landscape of PCa cells. By modifying the immune reactivity of PCa cells in culture, manipulation of *p*O_2_ can be proposed as a new avenue for improving diagnosis, prognosis and immunotherapy for PCa.

## Background

Prostate cancer (PCa) is the second most common cancer in men worldwide [1]. Metastatic PCa is not curable by surgery and is resistant to chemotherapy and ionizing radiation [2] eventually leading to death [3]. Similarly to most solid tumors, PCa tissues are characterized by low oxygen tension–hypoxia–that in PCa is a marker of poor clinical prognosis [4].

It has been generally accepted that microenvironmental hypoxia is among the reasons for resistance to treatment; it reduces the rate of cell proliferation and modulates numerous properties of cells known to promote resistance to chemotherapy and radiation [5–7]. In addition, hypoxia is associated with increased chromosomal instability, gene amplification, down-regulation of DNA damage repair pathways, and altered sensitivity to DNA damaging agents [8, 9] contributing thus to cancer phenotype.

Hypoxia triggers mechanisms that tend to reinstate oxygen homeostasis by changes in gene expression mediated mainly by the hypoxia-inducible factors (HIFs). HIFs are heterodimeric proteins, each composed of one of the three α subunits and the single β subunit resulting in HIF-1, HIF-2 and HIF-3 [10]. In hypoxia, HIF-1 and HIF-2 rapidly accumulate and translocate to the nucleus [11] to activate genes involved in endothelial cell proliferation, migration, permeability, survival, and response to radiotherapy and chemotherapy [12–15].

Changes in oxygen tension (*p*O_2_) modulate innate and adaptive immunity as well [16, 17], but it is less clear whether hypoxia contributes to the difficulties in developing effective cancer immunotherapy [18]. While most immunotherapy studies have focused onto boosting immunity (in the face of cancer–associated immunosuppression [19–21]), comparatively less attention has been paid to the role of hypoxia in modulating the expression of tumor–associated antigens (TAAs); this could be particularly germane for the vaccines consisting of whole cancer cells hitherto commonly prepared under standard (normoxic) tissue culture conditions [22]. Since *p*O_2_ values at standard culture conditions are much higher than those in hypoxic tumors, current normoxically cultured cell-based vaccines express antigens of unknown relevance to tumors in situ.

It has been reported recently that TAAs in human hypoxic glioma cells were more akin to TAAs in tumors in situ than to TAAs in cells cultured in air [23, 24]. The relevance of *p*O_2_ in modulation of immunogenicity of PCa cells is unknown; hence, we studied whether the TAAs in hypoxic PCa cells are different from normoxic PCa cells. Revealing oxygen-sensitive PCa-specific antigens could have important implications for development of better biomarkers and more effective immunotherapy for PCa.

## Methods

### Patients and plasma

All samples were collected retrospectively in the Mayo Clinic, Rochester, MN, USA and the University of Mississippi Medical Center, Jackson, MS, USA. The study protocol is in compliance with the Declaration of Helsinki and was approved by the corresponding Institutional Review Boards. Following surgical resection or donation, sample aliquots were prepared, immediately frozen at −80 °C, and stored until use. For identification of PCa-associated antigens in hypoxic PCa cell lysates and patient immune reactivity by 2-DE Western blot experiments, plasma from 25 early-stage PCa patients was utilized. Clinicopathological characteristics of PCa patients utilized in those experiments are summarized in Table 1. For the constitution of control groups, plasma provided by collaborators at Mayo Clinic, Rochester was obtained from 20 healthy age-matched volunteers, patients suffering from colon cancer (n = 10), lung cancer (n = 10), or rheumatoid arthritis (n = 20). For 2-DE Western blot protocols, pools (n = 5) of plasma samples were prepared and tested for nonspecific binding to the nitrocellulose membrane. To evaluate levels of autoantibodies specific for TAAs in human plasma by ELISA, additional plasma samples from healthy controls (n = 56) and patients with PCa (n = 29), obtained from the University of Mississippi Medical Center were included among the specimens described above. A group of plasma samples from 20 renal cell carcinoma patients, provided by collaborators at Mayo Clinic, Rochester was utilized as an additional control group. For ELISA protocols, plasma samples were individually assayed.

**Table 1:** Clinicopathological characteristics of prostate cancer patients utilized in 2-DE Western blot experiments

Age at surgery	
Number of cases	25
Mean (SD)	61.5 (4.58)
Median age, years (range)	61.0
Q1, Q3	58.0, 65.0
Range	(55.0–72.0)
Pre-op PSA	
Mean (SD)	5.8 (3.91)
Median age, years (range)	5.0
Q1, Q3	4.0, 6.3
Range	(1.7–20.5)
Clinical grade (GLEASON)	
Missing	0 (%)
6	24 (96 %)
7	1 (4 %)
8	0 (%)
9	0 (%)
Clinical T-stage, 1997 TNM	
Missing	0 (%)
T1C	16 (64 %)
T2a	9 (36 %)
T2b	0 (%)
T34	0 (%)
Pathologic grade (GLEASON)	
Missing	0 (%)
6	25 (100 %)
7	0 (%)
8	0 (%)
9	0 (%)
Pathologic stage, 1997 TNM	
Missing	0 (%)
T2aN0	8 (32 %)
T2bN0	17 (68 %)
T3aN0	0 (%)
TxN+	0 (%)
Treatment-prior to surgery	
Missing	0 (%)
No	25 (100 %)

### Cell culture

Cultured human PCa cell lines designated VCaP, LNCaP (American Type Culture Collection, Rockville, MD, USA), and C4-2B (Characterized Cell Line Core Facility, University of Texas MD Anderson Cancer Center) were seeded into T25 or T75 flasks (BD Falcon, Bedford, MA). LNCaP cells originate from a lymph node metastasis [25]. VCaP cells were generated from a vertebral metastatic lesion and harbor the TMPRSS2-ERG fusion (present in 40–60 % of PCa patients) [26, 27]. C4-2B cells represent a human bone metastatic PCa and are a derivative of LNCaP cells with more aggressive characteristics [28]. The interest in those cell lines is also based on their ample use by PCa researchers and the utilization of immortalized cells lines as components of heterologous whole cell PCa vaccines [29]. Cells were maintained in phenol-red supplemented RPMI-1640 (LNCaP and C4-2B) and DMEM (VCaP) medium containing 10 % heat-inactivated fetal bovine serum (Cellgro, Manassas, VA, USA) at 37 °C in humidified air enriched with 5 % CO_2_ and with O_2_ content either 20 % (approximately 20 kPa, normoxic), 2 % (approximately 2 kPa, hypoxic), or 1 % in some cases in a CO_2_ incubator (Binder, Germany). The cells were trypsinized at 80–90 % confluence and plated at the density of 25,000 cells/cm^2^ (LNCaP and VCaP) or 12,000 cells/cm^2^ (C4-2B). The medium was not refreshed during the course of the experiments. To evaluate cell viability, cells excluding trypan blue were counted by the aid of a hemocytometer.

### ELISA measurement of cytokines and autoantibodies

Concentrations of vascular endothelial growth factor (VEGF), a marker of hypoxia, were measured in conditioned culture media by ELISA (R&D Systems, Minneapolis, MN, USA). Levels of autoantibodies specific for heat-shock protein (HSP) 60 and HSP70 in human plasma were measured using IgG/A/M ELISA kits for HSP60 and HSP70, respectively (Enzo Life Sciences, Plymounth Meeting, PA, USA). Provided recombinant antigens were used as standards. Autoantibodies for heterogeneous nuclear ribonucleoprotein L (hnRNP L) were assayed using the pertinent ELISA kit (CUSABIO, Wuhan, China). The assays were conducted according to manufacturers’ guidelines. Plasma from healthy volunteers and patients with PCa, colorectal cancer, lung cancer, renal cell carcinoma, or rheumatoid arthritis were diluted thousand-fold. The cutoff reactivity for disease was defined as any value that was higher than the mean of the value plus twofold of standard deviation observed in normal plasma.

### Quantitative RT-PCR

Total mRNA was isolated using RNeasy Mini kit (Qiagen, Germantown, MD, USA) according to manufacturer’s instructions: One µg RNA was reversely transcribed using SuperScript III First Strand Synthesis (Invitrogen, Grand Island, NY, USA). Subsequently, quantitative PCR was performed with a LightCycler 480 SYBR Green I Master (Roche, Madison, WI). Levels of mRNA were normalized relative to the levels of control ribosomal protein S28 (RPS28) mRNA [30]. Data were analyzed by the Delta Delta Ct (2^−∆∆CT^) method using Excel program. Primer sequences used were (forward/reverse): VEGF-α: agtccaacatcaccatgcag/ttccctttcctcgaactgattt; and RPS28: ttttggagtcagagcgagaag/agcatctcagttacgtgtgg.

### Cell lysate preparation and labeling

LNCaP and VCaP cells grown in T75 flasks for four days and seven days, respectively, were washed with cold phosphate-buffered saline (PBS), scraped and centrifuged in PBS at 380×*g* for 5 min at 4 °C. Cells from up to 3 T75 flasks were resuspended in 1 mL PBS and centrifuged at 16,000×*g* for 1 min. To the pellet, 1.5 volume of 2D lysis buffer (7 M urea, 2 M thiourea, 4 % w/v CHAPS and 40 mM Tris, pH 8.5, 1 × nuclease mix; GE Healthcare, Pittsburgh, PA) was added together with 1x protease inhibitor cocktail set III (per mL of 2D lysis buffer containing 10 µL of 100x protease inhibitor cocktail set III; Calbiochem, San Diego, CA, USA). Cells were sonicated on ice for 30 min followed by continuous shaking for 45 min at 4 °C. Lysates were centrifuged at 24,000×*g* for 10 min. Supernatants were collected and saved at −80 °C. Protein concentration was determined by Bradford assay (Bio-Rad, Hercules, CA, USA) using bovine γ-globulin (Pierce, Rockford, IL, USA) as standard. Prior to 2-DE, 50 µg of lysate protein was labeled with 400 pM of differential in-gel electrophoresis (DIGE) fluor Cy5 minimal dye (GE Healthcare). Lysates were incubated with dyes for 30 min on ice in the dark. Labeling reaction was stopped by the addition of 1 μL of 10 mM lysine and incubation for 10 min on ice in the dark.

### Two dimensional gel electrophoresis (2DGE) and silver staining

Fifty µg protein per sample was diluted in 2D lysis buffer (without inhibitors) containing 30 mM DTT, 1 % 3–10 Pharmalyte ampholyte mixture and 0.25 % 3–10 non-linear (NL) immobilization pH gradient (IPG) buffer (GE Healthcare, Pittsburgh, PA, USA). After shaking for 30 min, the samples were dispensed into the isoelectric focusing tray, overlaid with 11 cm 3-10NL IPG strips and mineral oil, passively rehydrated for 11 h, and focused for a total of 35,000 Vh (Protean IEF Cell, Bio-Rad). After isoelectric focusing, the strips were immersed in equilibration buffer containing 1 % DTT for 10 min, followed by equilibration buffer with 2 % iodoacetamide for 15 min. The second dimension was carried out on Criterion 10 % gels (Bio-Rad) for 10 min at 140 V, followed by 1 h at 200 V. To detect the fluor Cy5–stained spots, the gel was placed directly between glass plates in a Typhoon 9410 variable mode imager (GE Healthcare) using 633-nm excitation and 670-nm emission wavelengths (optimal for detection of DIGE fluor Cy5). Additionally, electrophoresed proteins were visualized by silver staining. Images were analyzed and stained spots identified using PDQuest sofware (Bio-Rad) according to manufacturer’s protocols.

### 2D Western blotting

To identify PCa-associated autoantibodies, plasma samples were electrophoresed as described. Electrophoresed proteins were electro-transferred from the gel to nitrocellulose membranes (Bio-Rad) and blocked with pooled patient or normal plasma diluted 1/300 in blocking buffer. Subsequently the membrane was incubated with chicken anti-human IgG conjugated with HRP (diluted 1/3000 in blocking buffer; Abcam, Cambridge, MA, USA). After the addition of a chemiluminescent substrate (Thermo Fisher Scientific, Rockford, IL), membranes were immediately exposed on a CL-Xposure film (Thermo Fisher Scientific) and scanned with an Epson Perfection 4490 Photo scanner (Long Beach, CA, USA) for detection of spots.

### Protein digestion and mass spectrometry

Spots of interest identified by PDQuest were excised from gels, destained with 100 mM ammonium bicarbonate in 30 % acetonitrile until transparent and dried in a vacuum centrifuge. Proteins were proteolyzed with 25 ng of modified trypsin (Promega, Madison, WI, USA) in 25 mM ammonium bicarbonate at 37 °C overnight. Peptides were precipitated with 0.1 % trifluoroacetic acid and 60 % acetonitrile, vacuum-dried and analyzed by Ultraflex II MALDI-TOF system (Bruker Daltonics, Bremen, Germany). Spectra were analyzed by Biotools MS software (Bruker Daltonics) to perform peptide mass fingerprinting. We identified the proteins in the SwissProt database for Homo sapiens using carbamidomethyl on cystein as the fixed modification and methionine oxidation as variable modification.

### SDS-PAGE and Western blot of tumor tissue lysates

Protein extracts were prepared from frozen prostate tissue obtained from PCa patients (n = 8) and cystoprostatectomy patients (n = 4; used as control). Cysprostatectomy is a surgical procedure in which the urinary bladder and prostate gland are removed. The procedure combines cystectomy and prostatectomy and occurred in our situation for bladder cancer tumors. Tissues were homogenized in an IKA Work tissue homogenizer (Wilmington, NC, USA). Proteins were extracted from the homogenate with the AllPrep DNA/RNA/Protein Mini Kit (Qiagen, Germantown, MD) according to manufacturer’s guidelines. Thirty µg protein were resolved in a 10.5–14 % SDS-PAGE gradient gel, transferred to a nitrocellulose membrane and incubated with blocking buffer containing primary antibodies specific for HSP60 (diluted 1/250; Abcam), hnRNP L (1/5000; Abcam), and β-actin (1/5000; Santa Cruz Biotechnology, Santa Cruz, CA, USA). Bound primary antibodies were visualized with HRP-conjugated antibodies specific for human IgG (diluted 1/1000–5000 in blocking buffer; Abcam, Cambridge, MA, USA). After addition of a chemiluminescent substrate SuperSignal West Pico (Thermo Scientific, Rockford, IL, USA), the membrane was immediately exposed on a CL-Xposure film (Thermo Fisher Scientific) and scanned with an Epson Perfection 4490 Photo scanner to detect bands. Relative intensities of the bands were quantified using Image J Software (NIH online; Bethesda, MD); recorded values were normalized to the intensity of the respective β-actin signal.

### Statistics

All reported values represent three independent experiments expressed as mean ± SEM. Data for cell proliferation, VEGF release, and mRNA expression were analyzed by two-way ANOVA (*p*O_2_ vs. day), followed by a post hoc Student–Newman–Keuls multiple-comparisons test. Difference in HSP60 and hnRNP L proteins between tumor and normal tissue lysates were analyzed by the Mann–Whitney U test. Comparisons of plasma autoantibody levels for HSP60, HSP70 and hnRNP L among different patient groups were done using one-way ANOVA, followed by a post hoc Student–Newman–Keuls multiple-comparisons test (HSP60 and HSP70) or one-tailed *t* test (hnRNP L). A difference was considered significant at *p* < 0.05.

## Results

### PCa cells differ in sensitivity to hypoxia

**Fig. 1:**
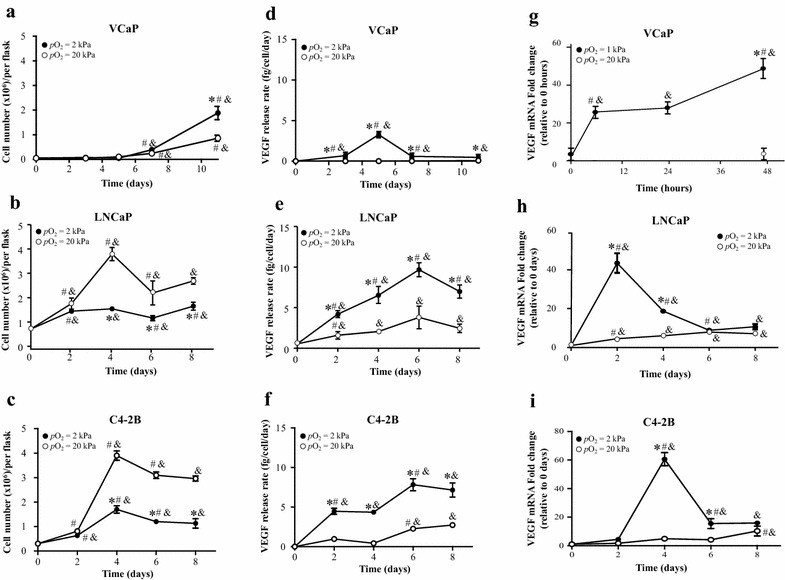
Effect of hypoxia on PCa cells. Effects on cell viability: VCaP (**a**), LNCaP (**b**) and C4-2B (**c**) cells were cultured at *p*O_2_ = 1 or 2 kPa (*filled circle*) or 20 kPa (*circle*). Alive cells were counted in triplicate flasks using trypan blue exclusion to differentiate dead cells. Effects on VEGF production: Rates of VEGF release in conditioned culture media were measured by ELISA for VCaP (**d**), LNCaP (**e**) and C4-2B (**f**) cells. Assessment of VEGF transcripts: mRNA levels of VEGF were analyzed in VCaP (**g**), LNCaP (**h**) and C4-2B (**i**) cells by qRT-PCR. *p < 0.05 when data at hypoxia is compared to values at normoxia O_2_ at the same time point; ^#^ *p* < 0.05 when data at hypoxia or normoxia is compared to a previous time point within the same *p*O_2_; ^&^ *p* < 0.05 when a specific data point is compared to the point at day 0 within the same *p*O_2_

To explore the effect of *p*O_2_ on cell viability of PCa cells, we exposed them to *p*O_2_ = 20 kPa or 2 kPa. Cells were counted as a function of time. The selection of cell lines utilized in this study is based on their distinct origin [25–27], representation of particular patients spectrum [25–27], and involvement as components PCa vaccines [29]. VCaP cells (Fig. 1a) showed a slow increase in cell numbers with significant increase from basal levels at day 7 (*p* < 0.05). Evaluation at day 11 evidenced twofold higher cell numbers in cells growing under hypoxia, relative to cells cultured in normoxic conditions (*p* < 0.05). Relative to VCaP cells, LNCaP cells (Fig. 1b) had a faster growth kinetics. Under normoxia, cells reached maximum numbers at day 4. At that time point, however, cells cultured in hypoxia had only close to a third of numbers relative those cultured in normoxic conditions (*p* < 0.05). Cell numbers did not experience further variation in LNCaP cultures under hypoxia. Similarly, C4-2B cells, the subline derived from LNCaP cells, had reduced viability when grown in *p*O_2_ = 2 kPa relative to that observed in cells growing at 20 kPa. Independently of oxygen levels, the maximum number of C4-2B cells in cultures cells was reached at day 4 (Fig. 1c). At that point, however, cells cultured in normoxic conditions had 2.3-fold more cells when compared to cells growing in hypoxia (*p* < 0.05). Cell numbers under normoxia and hypoxia did not experience further variation (compared to that observed at day 4) until completion of the experiment (day 10; *p* < 0.05).

*VEGF* is a hypoxia-inducible gene associated with the growth, aggressiveness and down-modulation of anti-tumoral immunity [31]. Assessment of the release rate of this pro-angiogenic factor to the conditioned media revealed distinct patterns of induction in PCa cells growing under hypoxia. VCaP cells (Fig. 1d) secreted VEGF at observable rates on day 3 (*p* < 0.05) reaching the maximum rate on day 5. This release rate was maintained up to day 11. In contrast, LNCaP cells cultured under low oxygen (Fig. 1e) had a steady increase of VEGF release rate until day 6. At that time point, a tenfold increase (*p* < 0.05) was observed relative to the values at day 0. Measured release of VEGF decreased to 7.5-fold at day 8. C4-2B cells cultured under normoxia (Fig. 1f) did not release VEGF until day 6. At that time point, nearly 2.5 pg/cell/day (*p* < 0.05) VEGF was released; that rate remained constant until day 8. Cells growing at *p*O_2_ = 2 kPa, however, released higher VEGF faster relative to their counterparts growing at *p*O_2_ = 20 kPa (*p* < 0.05). A maximum (8 pg/cell/day) was reached at day 6 (*p* < 0.05) and maintained at day 8 (*p* < 0.05). To assess the effect of transcriptional regulation on the observed release of VEGF, qRT-PCR was performed. Early induction of VEGF transcripts, nearly 45-fold in VCaP (Fig. 1g) and LNCaP (Fig. 1h) cells (at 2 days) and 60-fold in C4-2B (Fig. 1i) cells (*p* < 0.05), was observed in all cell lines when cultured under hypoxia. In cells growing under normoxia, VEGF transcripts were also induced (nearly tenfold in LNCaP and C4-2B cells, *p* < 0.05). The induction of VEGF in cells growing in normoxia, however, was observed at later time points and at a lesser extent in comparison to cells cultured under hypoxia. All together, these results indicate that, in accordance to reported findings [32, 33], PCa cells exposed to low levels of oxygen exhibit increased viability and elevated expression of the angiogenic factor, VEGF.

### Effects of hypoxia on immune reactivity of PCa cells

**Fig. 2:**
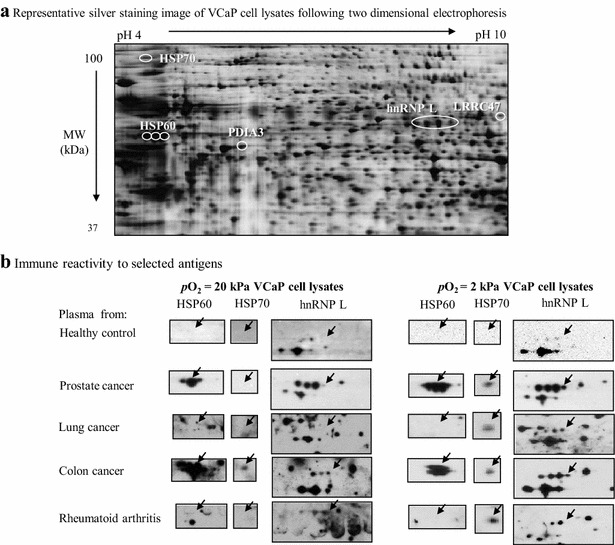
Identification of PCa-associated antigens in hypoxic VCaP cell lysates and patient plasma immune reactivity. VCaP cells were cultured at *p*O_2_ = 2 kPa and 20 kPa conditions for 7 days. Cell lysates were prepared and 50 μg proteins were loaded on pH 3–10 NL IPG strips for 2DGE. One set of gels was silver stained and other set was transferred to nitrocellulose membranes (**a**), incubated with pooled plasma from patients with PCa, colon cancer, lung cancer, rheumatoid arthritis (an autoimmune disease), and age matched healthy controls. Following incubation with chicken anti-human IgG-HRP, spots were identified by chemiluminescence (**b**). The *arrowheads* indicate protein spots of interest

**Fig. 3:**
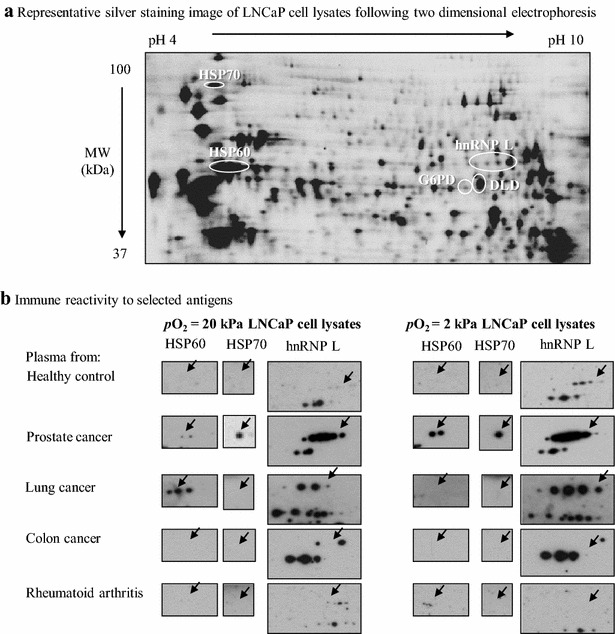
Identification of PCa-associated antigens in hypoxic LNCaP cell lysates and patient plasma immune reactivity. LNCaP cells were cultured at *p*O_2_ = 2 kPa and 20 kPa conditions for 4 days. Cell lysates were prepared and 50 μg proteins were loaded on pH 3–10 NL IPG strips for 2DGE. One set of gels was silver stained and other set was transferred to nitrocellulose membranes (**a**), incubated with pooled plasma from patients with PCa, colon cancer, lung cancer, rheumatoid arthritis (an autoimmune disease), and age matched healthy controls. Following incubation with chicken anti-human IgG-HRP, spots were identified by chemiluminescence (**b**). The *arrowheads* indicate protein spots of interest

To determine if *p*O_2_ modifies the reactivity of autoantibodies in CaP patient plasma, following published protocols [34] we harvested the cells at their highest reached cell density (VCaP, day 7; LNCaP, day 4), prepared lysates and resolved them by 2DGE. One of the two identical gels was silver-stained and the other blotted onto a nitrocellulose membrane. As antibodies reactive with tumors are commonly found in patient plasma [35], we pooled the plasma from PCa patients (n = 5/pool) and reacted it with the blotted proteins. For controls, plasma from healthy individuals, patients suffering from lung cancer, colon cancer or rheumatoid arthritis was used, n = 5/pool. We found that PCa-patient plasma consistently bound to more spots of VCaP (Fig. 2) and LNCaP (Fig. 3) cells than did control plasmas. In some cases, the size of spots differed significantly when PCa plasma or healthy control plasma was used. To assess the molecular identity of the spots reacted with PCa plasma only, we excised the selected spots, digested them by trypsin, and analyzed then by MALDI-TOF mass spectrometry. Use of DIGE fluor Cy5 minimal dye facilitated alignment of the silver-stained gel and the blotted membrane for spots selection. In VCaP cells we identified heat shock 70 kDa protein (HSP70), heat shock 60 kDa protein (HSP60), protein disulfide isomerase A3 (PDIA3), nuclear ribonucleoprotein (hnRNP L), and leucine-rich repeat-containing protein 47 (LRRC47) (Table 2). In LNCaP cells we identified HSP70, HSP60, hnRNP L, glucose-6-phosphate-1-dehydrogenase (G6PD), and dihydrolipoyl dehydrogenase (DLD) (Table 3).

**Table 2:** Potential PCa-associated antigens identified in VCaP cells

Protein name	FASTA accession number	MW (kDa)
Heat shock 70 kDa protein 4 (HSP70)	P34932	94.3
60 kDa heat shock protein (HSP60)	P10809	61.3
Protein disulfide isomerase A3 (PDIA3)	P30101	56.8
Heterogeneous nuclear ribonucleoprotein L (hnRNP L)	P14866	64.1
Leucine-rich repeat-containing protein 47 (LRRC47)	Q8N1G4	63.5

**Table 3:** Potential PCa-associated antigens identified in LNCaP cells

Protein name	FASTA accession number	MW (kDa)
Heat shock 70 kDa protein 4	P34932	94.3
60 kDa heat shock protein (HSP60)	P10809	61.3
Heterogeneous nuclear ribonucleoprotein L (hnRNP L)	P14866	64.1
Glucose-6-phosphate-1-dehydrogenase (G6PD)	P11413	59.2
Dihydrolipoyl dehydrogenase (DLD)	P09622	54.1

For independent confirmation we focused on three antigens, HSP60, HSP70, and hnRNP L. Regardless of the cell line used as source of antigens, healthy plasma reacted with normoxic lysates weakly and only faint spots or no spots for all three antigens was observed. The reactivity to PCa plasma was increased, particularly for HSP60 and hnRNP L, relative to healthy control plasma. Remarkably for all three analyzed antigens, interaction with hypoxic PCa cell lysates increased reactivity relative to that observed against normoxic cell lysates. For example (Figs. 2, 3), plasma reactivity for HSP60 was augmented 2 and eightfold in VCaP and LNCaP cells, respectively. Reactivity of PCa plasma to HSP70 using lysates obtained from VCaP cells grown *p*O_2_ = 20 kPa was not observed, but it was noted when lysates from cells grown at *p*O_2_ = 2 kPa were used as source of antigens. Similarly, a sevenfold increase in immune reactivity to HSP70 was observed in lysates from hypoxically-expanded LNCaP cells when compared to that noted for cells expanded under normoxia. hnRNP L (7 and twofold in VCaP and LNCaP cells, respectively), had increased reactivity to PCa plasma when hypoxic cell lysates were used.

Reactivity was also studied in other tumors. In particular, antibodies specific for HSP70 and hnRNP L were detected in the plasma of lung cancer patients only when reacting with VCaP cell lysates obtained from cells grown at *p*O_2_ = 2 kPa. Antibodies against HSP60, in a *p*O_2_ independent manner, were detected in the plasma of colon cancer patients (Fig. 2b). Autoantibodies specific for hnRNP L were detected at fourfold higher level in lung cancer plasma reacting with hypoxic LNCaP cell lysates, relative to the level found with normoxic lysates (Fig. 3b). Independent of the cell line used as source of antigens, plasma from rheumatoid arthritis patients did not have reactivity for the three analyzed antigens when cells were cultured under normal oxygen. Hypoxia, interestingly, allowed reactivity of rheumatoid arthritis patients’ plasma against HSP70 when lysates obtained from VCaP cells provided antigens. These results suggest that hypoxia increased reactivity of PCa cell lysates to patient plasma.

### Evaluation of plasma autoantibody levels of HSP60, HSP70, and hnRNP L

**Fig. 4:**
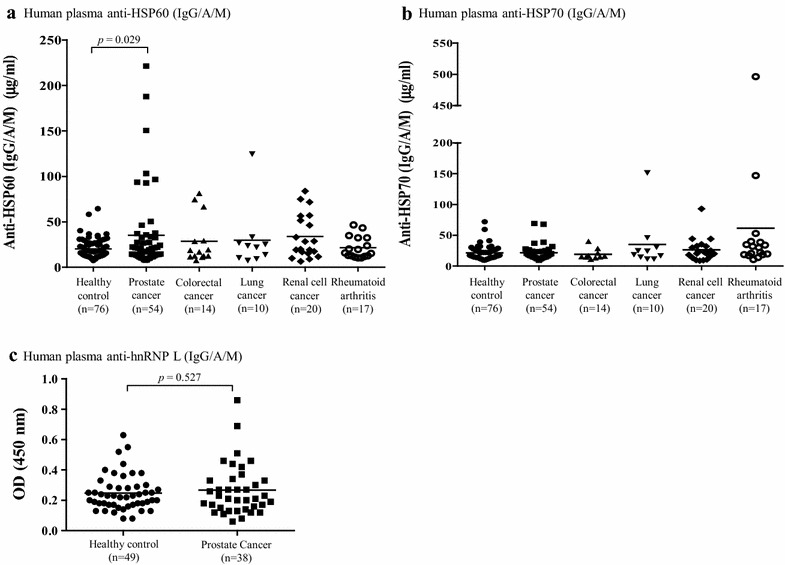
Levels of Hsp60, Hsp70 and hnRNP L autoantibody in human plasma. Autoantibodies to HSP60 (**a**), HSP70 (**b**) and hnRNP L (**c**) were quantitated in plasma by ELISA using recombinant antigens as standards. Healthy controls (*filled circle*) and patients with PCa (*filled square*), colorectal cancer (*filled triangle*), lung cancer (*filled inverted triangle*), renal cell carcinoma (*filled diamond*), and rheumatoid arthritis (*circle*). Plasma samples were diluted to 1/1000. The cutoff of reactivity was defined as the mean of sample plus twofolds of standard deviation from normal plasma. The level of significance was set at *p* < 0.05 between healthy control and PCa groups

Next, we set out to determine, at the individual patient level, the frequency of autoantibodies for HSP60, HSP70, and hnRNP L. For this purpose we performed ELISA tests. The assay allows for reproducible, accurate, and precise determination of IgG, IgA and IgM antibodies (total) in plasma. Recombinant human protein (e.g. HSP60, HSP70, and hnRNP L) were bound to the wells of the plate to bind anti-human antibodies for the analyte of interest present in plasma. The captured anti-human antibodies were detected with a HRP conjugated goat polyclonal antibody specific for human IgG, IgA, and IgM molecules. The frequency of HSP60 autoantibodies (Fig. 4a; Table 4) was 13.0 % (7/54) in PCa, 1.3 % (1/76) in healthy controls, 7.14 % (1/14) in colorectal cancer, 10.0 % (1/10) in lung cancer, 5.0 % (1/20) in renal cell carcinoma, and 0 % (0/17) in rheumatoid arthritis. Statistic difference was found only between PCa and healthy control groups (*p* < 0.05). These data suggest elevated HSP60 autoantibody levels in the plasma from PCa patients. The frequency of HSP70 autoantibodies (Fig. 4b; Table 4) was 5.3 % (2/38) in PCa, 3.7 % (2/54) in healthy controls, 7.14 % (0/10) in colorectal cancer, 20.0 % (2/10) in lung cancer, 5.0 % (1/20) in renal cell carcinoma, and 17.6 % (3/17) in rheumatoid arthritis. No statistic difference was found between control group and any cancer- or non-cancer group. The frequency of hnRNP L autoantibodies was 5.3 % (2/38) in PCa and 6.1 % (3/49) in healthy controls (Fig. 4c; Table 4). We did not evaluate hnRNP L autoantibodies in other diseases.

**Table 4:** Frequency of HSP60, HSP70 and hnRNP L autoantibodies in human plasma

Human plasma	*HSP60*			*HSP70*			*HnRNP L*		
	N	mg/ml (mean ± SD)	Positive cases (% of total cases)	N	mg/ml (mean ± SD)	Positive cases (% of total cases)	N	OD (mean ± SD)	Positive cases (% of total cases)
Prostate cancer	54	34.7 ± 43.9	7 (13 %); *P* < *0.05 compared to healthy controls*	38	21.8 ± 13.1	2 (5.3 %)	38	0.267 ± 0.168	2 (5.3 %)
Healthy control	76	23.0 ± 25.8	1 (1.3 %)	54	21.1 ± 11.8	2 (3.7 %)	49	0.248 ± 0.117	3 (6.1 %)
Colorectal cancer	14	28.7 ± 25.5	1 (7.1 %)	10	19.0 ± 8.8	0 (0 %)	–	–	–
Lung cancer	10	29.7 ± 34.4	1 (10.0 %)	10	34.9 ± 42.2	2 (20 %)	–	–	–
Renal cell carcinoma	20	33.8 ± 24.2	1 (5.0 %)	20	26.4 ± 19.0	1 (5.0 %)	–	–	–
Rheumatoid arthritis	17	21.5 ± 11.9	0 (0 %)	17	61.5 ± 116.3	3 (17.6 %)	–	–	–

### Higher levels of hnRNP-L proteins in PCa tissues

**Fig. 5:**
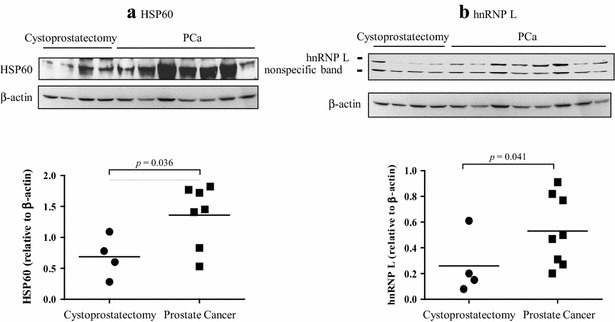
Expression of HSP60 (**a**) and hnRNP L (**b**) proteins in PCa tissues. Protein lysates extracted from frozen prostate tissues (8 samples from PCa patients and 4 samples from cytoprostatectomy patients) were separated by SDS-PAGE. HSP60 and hnRNP L levels in the samples were identified by Western blot as described in the “Methods”. The level of significance was set at *p* < 0.05 between PCa and cytoprostatectomy samples

To test the possibility that the immune reactivity of selected PCa-associated antigens in plasma was due to elevated expression of PCa-specific antigens in tumor tissues, we analyzed levels of HSP60 and hnRNP L in tumor tissue lysates using Western blot. Protein lysates were prepared from prostate tissues obtained from patients suffering PCa or cystoprostatectomy (controls). Separated proteins were transferred onto a nitrocellulose membrane and blotted against anti-human HSP60 and hnRNP L monoclonal antibodies. The level of HSP60 (Fig. 5a) and hnRNP L (Fig. 5b) proteins in PCa tumor tissues was close to twofold higher (*p* < 0.05), compared to control prostate tissue. These data suggest that protein expression for the selected potential PCa-associated antigens can be elevated in PCa tumors at the protein level.

## Discussion

We investigated the effect of hypoxia on the antigenic landscape in commonly used human PCa cell lines. As expected, hypoxia modified cell viability and promoted the release of the pro-angiogenic factor, VEGF. Use of a serologic approach and combined 2DGE expression profiling of PCa cells and Western blot with PCa patient plasma allowed identification of autoantibodies to PCa-associated antigens. Hypoxia increased the immune reactivity of plasma to PCa cell lysates. Some of these, hypoxia-reactive, TAAs were identified. In particular, we identified HSP60 as a valuable TAA with potential as a tumor marker in PCa tissues. Overall, our studies suggest that tumor–associated hypoxia is a relevant determinant of tumor antigenicity.

Low *p*O_2_ is commonly associated with tumor aggressiveness. For instance, hypoxia impacts the proliferation of rapidly-growing malignant tumors in vivo [36] and in vitro [37]. Although there were no effects on cell viability during the first 4 days in LNCaP and its derivative, the subline C4-2B, the cells in the hypoxic condition grew slower than the corresponding cells under normoxia (Fig. 1b, c). The result is consistent with previous reports in literature showing that LNCaP and PC-2 cells are less viable under hypoxia in comparison to normoxia [38, 39]. VCaP cells (Fig. 1a), on the contrary had higher cell numbers relative to cells cultured under normoxia. To date, no evaluation of the effects of low oxygen on the proliferation of VCaP has been reported. Our results therefore, are the first to suggest that hypoxia confers a survival advantage to the vertebral bone metastasis-derived, VCaP cells. In additional experiments (data not shown), when cultured in low oxygen, the brain metastasis-derived DU145 [40] and SV40-immortalized prostatic epithelial, P4E6 cells, derived from a primary prostate cancer biopsy [41], had reduced viability relative to cells growing in normoxia. On the contrary (data not shown), OnyCap23 cells, a subclone of the established prostate cell line PNT2-C2 [42] that was originally derived from a biopsy of a normal prostate gland, showed increased viability when cultured in hypoxic conditions. Of note, P4E6, OnyCaP23 and LNCaP cells have been previously utilized as components of an allogeneic whole cell PCa vaccine, partially successful in suppressing progression of the disease [22]. One limitation of our study is the unavailability of data generated using other cell lines besides those from prostatic carcinoma origin as positive of negative controls for hypoxia induction. Inclusion of these cell lines would allow assessment of relevant aspects such as the relative extent of the response to oxygen in PCa cells and the disease-related specificity of our results. These differences, attributed to cell-specific manners to respond to changes in metabolism and survival environment, should be considered when seeking improvement of allogeneic PCa vaccines.

As a critical component of the response of cancer cells to hypoxia, we analyzed the expression of VEGF, involved in the tumor growth and progression [43]. Low oxygen within the tumor microenvironment upregulates VEGF [44], and therefore this pro-angiogenic factor can be used as a marker of hypoxia. Particularly, in PCa cells, VEGF is reported to be expressed and secreted at high levels under hypoxic conditions [33, 45, 46]. In consistency with these previous reports, our results showed that, independent of the cell line used as source of VEGF, hypoxia enhanced VEGF release in PCa cells. This change, related to sustained increase >40–60 fold VEGF mRNA expression over basal levels (Fig. 1g–i), reflects the described [33, 45, 46] transcriptionally driven, induction of this pro-angiogenic factor under hypoxia. Similarly to VEGF, transcripts for hypoxia-inducible enzyme carbonic anhydrase 9 (CA9), overexpressed in cancer cells [47] and proposed as a useful marker for hypoxic exposure and tumor aggressiveness [48] were assessed by us (data not shown). When measured, the transcripts of CA9 were 150- and fivefold higher in hypoxic compared to normoxic VCaP cells and LNCaP, respectively. Altogether, our findings indicate that, in accordance to reported findings [49], PCa cells exposed to low levels of oxygen exhibit features associated with increased aggressiveness.

2DGE in combination with Western blot is a well-established method to identify antigens [50]. In the present study, we used this approach to separate and identify potential PCa-TAAs in whole cell lysates. As shown in Tables 2 and 3, the screening method demonstrated several protein spots that were specifically revealed by antibodies present in plasma from PCa patients. These proteins can be divided into two main categories based on their functions, i.e., metabolic enzymes (G6PD, and DLD) and molecular chaperones (HSP60, HSP70). Other candidate antigens included PDIA3 and hnRNP L. The metabolic requirements of cancer cells differ from that of their normal counterparts. To support their growth and proliferation, cancer cells adapt their metabolic processes to drive macromolecular biosynthesis [51, 52]. Therefore it is not surprising to see immune reactivity to metabolic enzymes in cancer patients. For example, higher level of G6PD [53] have been reported in sera and/or tumor tissues from several cancer patients. Further, these metabolic enzymes have been recognized as markers for TAA [54–58]. In all, our findings add to the reported findings and support the relevance of metabolic enzymes as members of the tumor-specific antigenic landscape. PDIA3 catalyzes oxidative protein folding and has a variety of effects on the yield of native lysozyme during the oxidative refolding of the reduced, denatured protein [59, 60]. Although up-regulation of PDIA3 was reported in breast cancer [34, 61] and hepatocellular carcinoma [62] tissues, to the best of our knowledge, it is the first time for detection of PDIA3 antibodies in plasma from PCa patients.

Among the identified TAAs, HSP60 and HSP70 were expressed in the studied PCa cell lines. HSPs are a group of highly evolutionary conserved proteins, including constitutively and inducible protein chaperones [63]. Mammalian cells express HSPs in response to heat as well as many other stress stimuli, including hypoxia [64]. Because HSPs play essential roles in tissue homeostasis [65], it has been postulated that they contribute to the development of many diseases, including tumors [66–68]. Up-regulated expression of HSPs has been reported in several cancers, including PCa [69–75]. Consistent with 2DGE and Western blot analysis, ELISA showed a higher level of anti-HSP60 IgG/A/M in plasma from PCa patients than that from healthy controls (Fig. 4a; Table 4). These results suggest that HSP60 is a TAA in PCa and are in agreement with reported findings showing HSP60 autoantibodies in sera from patients with breast cancer [34], colorectal cancer [76], and osteosarcoma [77]. Our data represent the first ones to suggest a specific humoral response against HSP60 in CaP. Further data analysis in an independent validation group is needed to evaluate the performance and diagnostic value of HSP60 autoantibodies.

HSP70 is expressed only at low or undetectable levels in most normal cells and tissues, but its expression is rapidly elevated by a variety of physical and chemical stressors [78]. Although there are many publications regarding the diagnostic, prognostic, and predictive implications of HSP70 in breast and several other cancers, its role in PCa remains unclear [79]. The value of HSP70 as PCa-associated antigen was suggested by our TAA screening, but it was not successfully validated by ELISA. This result contrasts with previous findings suggesting HSP70 as a potential TAA in esophageal carcinoma [80] and hepatocellular carcinoma [62, 81]. In our hands, just like in the findings from Cornford and collaborators [74], HSP70 was unaltered in the plasma of early stage PCa patients when compared with nonneoplastic prostatic epithelium. Regardless of these results, the authors indeed reported diminished expression of HSP70 in morphologically advanced cancers. Our report adds to the published information [74] suggesting that patterns of HSP70 are not significantly altered in early stage PCa patients (used in our studies) and may be, therefore, differentially modulated as a function of disease progression.

Heterogeneous nuclear ribonucleoprotein L is an abundant RNA-binding protein implicated in many bioprocesses [82], including those related to control of the expression of hypoxia-associated genes. For example, a specific interaction of hnRNP L with VEGF mRNA is thought to play an important role in hypoxia-induced post-transcriptional regulation of human VEGF mRNA stability [83]. Several lines of evidence show that hnRNP L is involved in tumorigenesis. For instance, phosphorylated hnRNP L promotes expression of the antiapoptotic form of caspase-9 (an initiator caspase) and thereby contributes to tumorigenesis [84, 85]. In PCa cells hnRNP L acts in mitosis as a src-associated 68 kDa (Sam68)-interacting protein and regulates splicing in response to signaling cascades [86]. In the present study, autoantibodies specific for hnRNP L were detected in PCa plasma reacting both with VCaP (Fig. 2) and LNCaP (Fig. 3) cell lysates. ELISA analysis, however, did not reveal difference in plasma hnRNP L autoantibody levels between PCa and the healthy control groups (Fig. 4c; Table 4). Regardless of these results, evaluation of the levels of hnRNP L in tumor tissue lysates using Western blot revealed that along with HSP60, protein level of hnRNP L in a small number of PCa tumor tissues was twofold higher compared to control prostate tissue (Fig. 5). These data trace back to tumor expression of HSP60, the TAA validated in plasma. Additionally, the results suggest that, despite the lack of relevant immune plasma reactivity, protein expression of hnRNP L in the tumor holds potential as marker and tumorigenic factor in PCa. Furthermore, this finding illustrates the importance of validation in the assessment of tumor markers. Since selection of proper relevant antigens is key for the generation of target-specific cancer vaccines, we think our results suggest that hn-RNP L may not be a good antigen to further pursue. Additional work, however is needed to settle the discussion.

Our analysis consistently revealed increased plasma reactivity when lysates from PCa cells cultured under hypoxia were used as source of antigens. These findings, despite of the limitation of lacking control lysates obtained from cells from non-prostatic origin, underscore relevant concepts. On one side, they sustain the reported immunogenic nature of PCa tumors [87] initiators, in this case of autoimmune responses against prostate antigens. On the other side, the results suggest a role of hypoxia as an immunostimulatory factor with potential to expand the antigenic landscape of clinically relevant TAAs for diagnosis and treatment of PCa. In this study, plasma of early stage, newly diagnosed PCa patients was utilized as source of TAAs. This was done in order to avoid confounding effects of advanced disease or treatments on immune reactivity [88]. As source of antigens we used cell lines of metastatic origin. It is certainly possible that those cell lines provided an antigenic landscape that may be different from that recognizable in plasma from newly diagnosed patients. This is another limitation of our experimental approach. Future studies should be focused on validating the potential of the identified TAAs. This should be done in scenarios including disease progression and/or established or experimental therapeutic modalities. Critical questions needing answer in terms of determinants of tumor immunogenicity, or the ability of the immune system to induce adaptive responses to tumors, pertain to the upstream tumor specific mediators that initiate the cascade of events that lead to immunosuppression [89]. Tumor hypoxia, represents an important impediment to immune-mediated control of tumor growth [5]. Our results indicate that hypoxia increases detection of humoral autoimmune responses. The involvement of hypoxia in actively eliciting tumor immunity or playing and adjuvant role to elicit more effective immune responses is, however, unknown. In favor of this role, recent characterization of the immune responses in a murine model of glioma, led to the identification of oxygen as an “immunologic switch” affecting both cell-mediated and humoral immune responses elicited by tumor cell lysates [23]. These data, further validated using tumor lysates from patients obtained under hypoxic culture as source of the antigenic repertoire [24], demonstrate the ability of low oxygen to enhance immunogenicity. Our results, supported by the reported findings [23, 24], allow us to propose that PCa vaccines made by expansion of primary tumor cells in low oxygen will enhance cell-based therapeutic vaccines for PCa. This premise deserves further investigation. Utilization of three dimensional culture systems such as matrigel or spheroids [33] and patient-derived primary PCa tumors as source of antigens, or implementation of an animal model to evaluate immune responses in vivo are necessary to validate our proposal. Use of a 2-dimensional culture system is a limitation of our study. Certainly the use of microenvironment-relevant experimental conditions would allow improvement in the identification hypoxia-specific tumor-associated antigens. We focused our studies in PCa cell lines and use of 2-dimensional cultures because those conditions have been utilized previously to generate allogeneic PCa vaccines with limited beneficial effects in patients [18, 22, 41]. This decision was based on the rationale of providing the scientific community with the proof of principle directing future studies geared into the improvement of current vaccines. Nevertheless, the tumor microenvironment-dependency of our findings is to be proven in future studies. Use of relevant experimental setups will allow us to demonstrate the ability of low oxygen to enhance immunogenicity or its ability to enhance cell-based therapeutic vaccines for PCa.

LNCaP and VCaP cells showed differences in expression of PCa-associated antigens. For instance, while HSP70, HSP60, hnRNP L and snRNP70 antigens were detected in both hypoxic cell lines, PDIA3 and LRRC47 antigens were found only in hypoxic VCaP cells. Likewise G6PD, PHGDH, ALA and DLD were specifically detected in hypoxic LNCaP cells. The origin of these findings is based on the distinct nature of the cell lines utilized in our studies; LNCaP cells are originated from a lymph node metastasis [25] and VCaP cells are generated from a vertebral metastatic lesion and harbor the TMPRSS2-ERG fusion (present in 40–60 % of PCa patients) [26, 27]. Our results implicate that the cell-specific spectrum of PCa-associated antigens may be relevant for development of more effective individualized prognostication and allogeneic cell-based immunotherapy. This statement is particularly relevant in the light of the high complexity, heterogeneity, and immune escape inherent to PCa [90].

## Conclusions

The role of *p*O_2_ in tumor biology has been unappreciated. Recently, tumor–associated hypoxia has been associated with malignant progression, metastasis, resistance to therapy, and poor clinical outcome. Our results validate the relevance of tumor-associated hypoxia in CaP immune-reactivity. More importantly, our findings define the potential of hypoxia as a tool in the development of markers and immunotherapeutic targets for PCa.

## Abbreviations

CA9: carbonic anhydrase 9
DLD: dihydrolipoyl dehydrogenase
G6PD: glucose-6-phosphate-1-dehydrogenase
HSP: heat-shock protein
hnRNP L: heterogeneous nuclear ribonucleoprotein L
HIFs: hypoxia-inducible factors
LRRC47: leucine-rich repeat-containing protein 47
PO_2_: oxygen tension
PCa: prostate cancer
PDIA3: protein disulfide isomerase A3
TAAs: tumor-associated antigens
2DGE: two dimensional gel electrophoresis
VEGF: vascular endothelial growth factor
